# Supplementary material for: Controlled tumor heterogeneity in a co-culture system by 3D bio-printed tumor-on-chip model
Source: Sci Rep. 2023 Aug 22;13:13648. doi: 10.1038/s41598-023-40680-x (PMC10444838; doi:10.1038/s41598-023-40680-x)
Supplement: Supplementary file 1 — Supplementary Information. [file 41598_2023_40680_MOESM1_ESM.docx]

**Supporting Information**

**Controlled tumor heterogeneity in a co-culture system by 3D bio-printed tumor-on-chip model**

**Nafiseh Moghimi,^1,4^ Seied Ali Hosseini,^2^ Altay Burak Dalan ^1,3^* Dorsa Mohammadrezaei,^1^ Aaron Goldman,^4^ Mohammad Kohandel^1^***

*Department of Applied Mathematics, University of Waterloo, Waterloo, Canada,^1^ Electrical Engineering Department, University of Waterloo, Waterloo, Canada,^2^ Department of Medical Genetics, School of Medicine, Yeditepe University, Istanbul, Turkey,^3^ Department of Medicine, Harvard Medical School, Boston, MA, USA; Division of Engineering in Medicine, Brigham and Women's Hospital, Boston, MA, USA^4^*

**correspondence to:* *nmoghimi@uwaterloo.ca*

**Rheological properties:**

The rheological properties of hydrogel samples for four combinations of A1G4, A1G8, A4G4 and A8G4, (which A1G4 represents Alginate 1%, Gelatin 4%, etc) with the best printability have been studied. Figure 2 shows the rheology characterization of hydrogel mixtures. Figure 2A, shows the storage modulus (G’) and loss modulus (G”) obtained at room temperature by frequency oscillation measurement. The viscoelasticity data for all samples show a higher storage modulus than loss modulus, which indicates the gel-like property of the material. Both storage and loss modulus were found to increase as frequency increased.

Tan δ values were calculated and are shown in Figure 2B versus frequency for four hydrogels. Tan δ values were found <1, which suggested that all the hydrogel mixtures are more gel like mixture than liquid like. The strength of hydrogel was increased as alginate concentration increased up to alginate 4% and dropped when alginate concentration is 8%.

The viscosity of all hydrogels mixtures, shown in Figure 2C, decreased with increasing shear rate and the shear-thinning behavior was observed for all four samples. The viscosity curves of all the hydrogel mixtures showed a similar pattern which suggests that all the hydrogels had shear thinning behaviors. The loss and storage modulus at 40 Hz are illustrated in Figure 2D. A4G4 shows the highest storage (elastic) modulus. It has also been observed that 4% gelatin contributes a profound viscoelastic property when mixed with 4% alginate, which resulted in consistent extrusion through the nozzle.


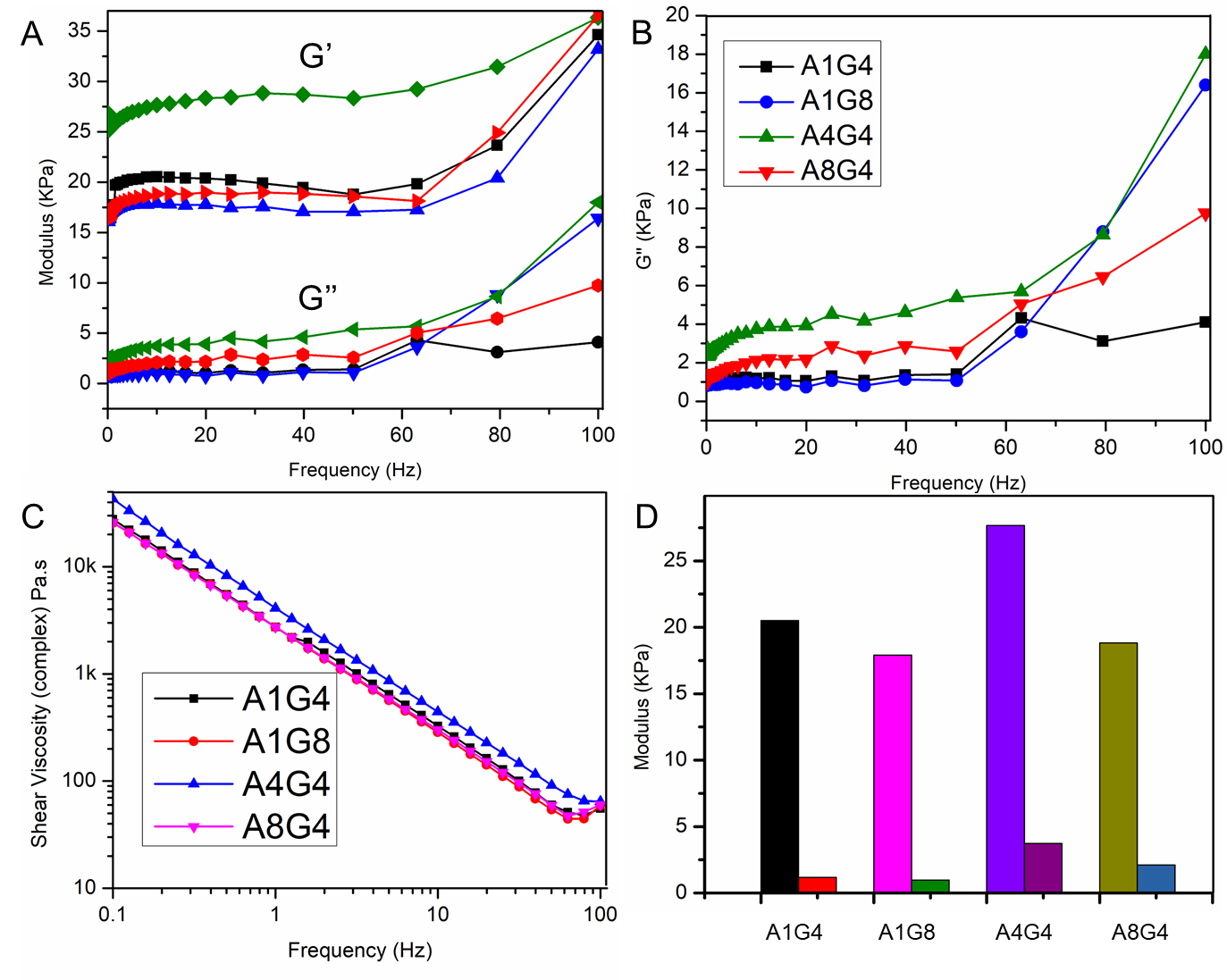


**Figure S1:** Rheology characterization of hydrogel mixtures with A1G4, A4G4, A8G4 and A1G8 (1, 4, 8, and 1 % alginate and 4, 4, 4, and 8% gelatin respectively). (A) Storage and loss modulus of A1G4, A1G8, A4G4 and A8G4 under different oscillatory frequencies. (B) Tan δ vs frequency of the hydrogel mixtures. Tan δ value were found <1 for all hydrogel mixtures. (C) Viscosity of hydrogels at different shear rate. Viscosity of all hydrogels decreased with increasing shear rate. (D) Histogram of modulus for different hydrogel mixtures. A4G4 shows the highest storage modulus. (D) loss modulus, G”

**Supplementary Table 1**

| **Bioink** | **Pressure, Gauges (22G Nozzle)** | **Filament** | **Printability** |
| --- | --- | --- | --- |
| **Alg 1%/ Gel 4% (A1G4)** | 20-30 KPa | Has nods with red nozzle, spreads with blue nozzle | Could not hold the structure, especially with blue nozzle |
| Alg 1%/ Gel 5% (A1G5) | 20-30 KPa | Has nods with red nozzle, spreads with blue nozzle | Could not hold the structure, especially with blue nozzle |
| **Alg 4%/ Gel 4% (A4G4)** | 60-70 KPa | smooth | Accurate printing |
| **Alg 8%/ Gel 4% (A8G4)** | 100-110 KPa | Very sharp and smooth | Accurate printing |
| Alg 4%/ Gel 8% (A4G8) | 130-140 KPa | Has nods | Lack of printing accuracy, too dense and difficult to print |
| **Alg 1%/ Gel 8% (A1G8)** | 70-80 KPa | smooth | Accurate printing |
| Alg 1%/ Gel 10% (A1G10) | 100-110 KPa | Has nods | Lack of printing accuracy |


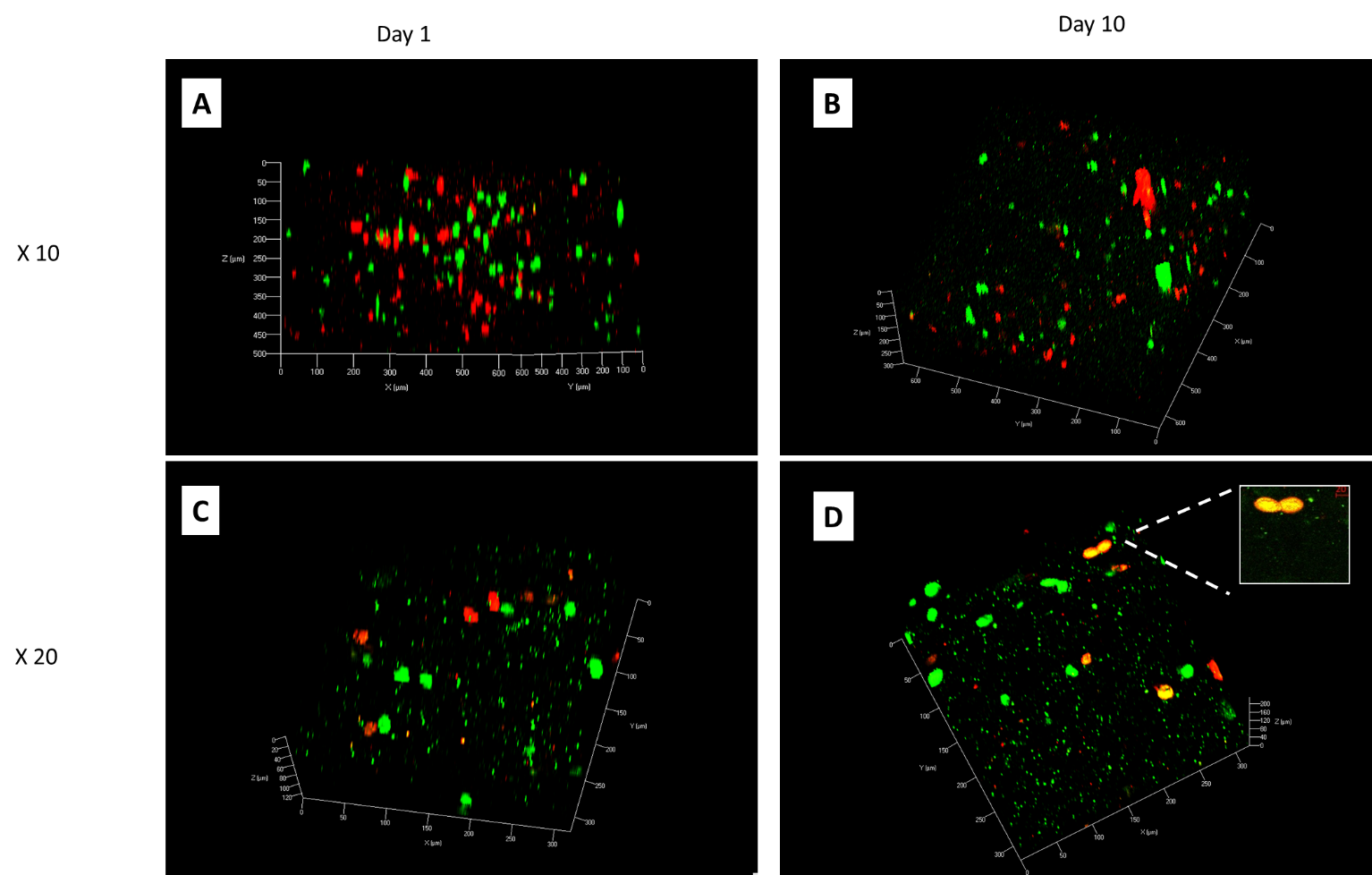


**Figure S2**: Confocal image of the co-culture construct at (A) day 1, (B) day 10. (C) and (D) are the higher magnification of two different layer of the construct at day 10. MDA-MB-231 cells marked as green and MCF marked as red.
